# Construct validity of questionnaires for the original and revised reinforcement sensitivity theory

**DOI:** 10.3389/fpsyg.2022.1026894

**Published:** 2022-11-21

**Authors:** Anja Leue, Martin Reuter, Philip J. Corr, Ulrich Ettinger

**Affiliations:** ^1^Institute of Psychology, University of Kiel, Kiel, Germany; ^2^Department of Psychology, University of Bonn, Bonn, Germany; ^3^Institute of Psychology, University of London, London, United Kingdom

**Keywords:** conflict monitoring, trait-BIS/BAS, CFA, item parceling, construct validity

## Abstract

This study highlights psychometric properties and evidence of construct validity on parcel-level for questionnaires on the original and revised reinforcement sensitivity theory. Our data (*N* = 1,076) suggest good to very good psychometric properties and moderate to excellent internal consistencies. Confirmatory factor analysis (CFA) models suggest a very good model fit for the first-order, four factor models of the Carver-White BIS/BAS scales, Reinforcement Sensitivity Theory – Personality Questionnaire (RST-PQ), the two-factor model of revised Reinforcement Sensitivity Theory-Questionnaire (rRST-Q) and for the bifactor model of the Conflict Monitoring Questionnaire (CMQ-44). The CMQ-44 extends the psychometric measurement of previous trait-(r)BIS and trait-BAS scales. Factor scores of CMQ-44 cognitive demand correlate positively with factor scores of Carver-White BIS and all Carver-White BAS subfactors except RST-PQ-Impulsivity suggesting that CMQ-44 cognitive demand addresses Carver-White trait-BIS specifically and more generally the trait-BAS core. CMQ-44 anticipation of negative consequences and response adaptation correlate negatively with trait-BAS, whereas the second-order factor performance monitoring extends the rRST trait-space and correlates positively with trait-BAS.

## Introduction

The reinforcement sensitivity theory (RST, Gray, 1987) and its latest revision in 2000 (rRST, Gray and McNaughton, 2000) have motivated a number of questionnaire developments in English starting more than 30 years ago (Wilson et al., 1989, 1990) but also in recent years (Corr, 2008; Corr and Cooper, 2016). One of the most important implications of rRST compared to former RST versions is its differentiation of the functioning of the behavioral inhibition system (BIS), the behavioral approach system (BAS), and the Fight-Flight-Freeze system (FFFS). In rRST, the BIS is presumed to detect and solve conflicts that have implications for behavioral adaptations like BAS-related and/or FFFS-related approach behavior (e.g., Fight) and FFFS-related withdrawal behavior such as Flight or Freeze (Corr, 2008). That is, in case of conflicting information the BIS changes from checking (e.g., observing, comparing mode) to control mode by innervating behavioral changes of the BAS and the FFFS (Gray and McNaughton, 2000). The abbreviation “rRST” is used to indicate that scales of the “newer” rRST (Gray and McNaughton, 2000; Corr, 2008) are discussed or compared to scales of the “older” RST (Gray, 1987).

The present study incorporates a construct validation of rRST questionnaires in German language that have been published between 2015 and 2021 (Reuter et al., 2015; Pugnaghi et al., 2018; Leue and Beauducel, 2021). For the purpose of comparison, the construct validation of the more recent rRST questionnaires in this study also includes the German version of the Carver and White (1994) BIS/BAS scales developed based on the “older” RST (Strobel et al., 2001). The number of RST-related personality questionnaires developed in different languages between the 1980s and 2021 is larger than presented here (see Corr, 2008; Krupić et al., 2016; Walker and Jackson, 2016; Leue and Beauducel, 2021, for further questionnaires). Among psychometric studies, different models for disentangling rRST scales have been tested using confirmatory factor analysis (CFA). Therefore, the aim of the present study is four-fold: (1) We highlight the psychometric properties (i.e., item means, part-whole corrected item-total correlations, different types of reliabilities) and descriptive statistics. (2) We present evidence of factorial validity for different trait-models of the (r)RST questionnaires. (3) We describe whether (r)RST latent factors are measurement-equivalent across gender. (4) We aim at presenting *a priori* predicted convergent and discriminant construct validity (Campbell and Fiske, 1959) based on factor scores.

### Psychometric properties and confirmatory factor analysis of (r)RST trait-scales

For all (r)RST-related questionnaires examined in this study, psychometric properties have been reported as a part of the construct validation (Strobel et al., 2001; Reuter et al., 2015; Pugnaghi et al., 2018; Leue and Beauducel, 2021). In terms of published criteria, all (r)RST questionnaires in the present study reveal good to excellent psychometric properties (i.e., positive and ≥0.10 part-whole corrected item total correlations) and good to excellent internal consistencies (Nunnally and Bernstein, 1994). Therefore, the present study seeks to investigate psychometric properties of the (r)RST questionnaires in relation to conceptual replication issues (research question 1).

The Carver and White BIS/BAS scales (Carver and White, 1994) with their German translation (Strobel et al., 2001) measure trait-BIS as a sensitivity to aversive reinforcement (Gray, 1987). Moreover, the Carver and White BIS/BAS scales assess trait-BAS as a total scale. The total trait-BAS scale incorporates three BAS subscales entitled as BAS-Drive, BAS-Reward Responsiveness, and BAS-Fun Seeking. FFFS-related behavior has not been psychometrically assessed in the Carver and White BIS/BAS scales. The Reuter-Montag Reinforcement Sensitivity Questionnaire (Reuter and Montag’s rRST-Q, Reuter et al., 2015) includes trait-BAS, trait-BIS and trait-FFFS in accordance with rRST. Similarly, the Reinforcement Sensitivity Theory – Personality Questionnaire (RST-PQ, Corr and Cooper, 2016) and its German translation (Pugnaghi et al., 2018) measures trait-BIS, trait-FFFS, and trait-BAS in terms of rRST. Trait-BIS and trait-FFFS are conceived as “unitary defensive factors” (Pugnaghi et al., 2018, p. 2) including four and three subscales, respectively. Trait-BAS incorporates four subscales. Table 1 summarizes short definitions of the trait-BIS, trait-BAS and trait-FFFS scales.

**TABLE 1:** Summary of scale descriptions.

Questionnaires	Scale	Description
**Questionnaires with Trait-BIS, Trait-BAS related scales**		
Carver-White (CW) BIS/BAS scales	CW BIS (“old” RST)	Strobel et al. (2001): Development and experience of negative emotions like frustration, sadness and depression.
	CW BAS (“old” RST)	Strobel et al. (2001): Approach behavior and active avoidance behavior following conditioned signals of reward and relief of punishment.
	CW BAS-Fun Seeking	Carver and White (1994, p. 322): “…desire for new rewards and a willingness to approach a potentially event on the spur of the moment.”
	CW BAS-Reward Responsiveness	Carver and White (1994, p. 322): “…positive responses to the occurrence or anticipation of reward.”
	CW BAS-Drive	Carver and White (1994, p. 322): “…Persistent pursuit of desired goals.”
Conflict-Monitoring-Questionnaire-44/28 (CMQ-44/28)	Cognitive demand	Leue and Beauducel (2021, p. 2): “…serves as an aversive teaching signal that intensifies the conflict monitoring.” We refer to Botivinick’s conception of “effort” as a conceptual basis for varying conflict monitoring intensity (Botvinick, 2007; Botvinick and Rosen, 2009).
	Anticipation of negative consequences	Leue and Beauducel (2021, p. 2): “In case of conflicting or incompatible information that can be induced by (the anticipation) of negative feedback the checking mode of the BIS switches into a control mode.”
	Response adaptation	Leue and Beauducel (2021, p. 3): “…intense and rapid adaptation of responses” as required in go/nogo tasks.
	Uncertainty of reinforcement	Leue and Beauducel (2021, p. 3): “… intense experience of uncertainty of reinforcement during decision making” because of incompatible alternatives as required in discrimination learning tasks (see also Smith et al., 2019, p. 1883).
**Questionnaires with Trait-BIS, Trait-BAS, Trait-FFFS scales**		
Reinforcement Sensitivity Theory – Personality Questionnaire (RST-PQ)	Trait-BIS	Corr and Cooper (2016, p. 1429): BIS resolves conflicts until behavioral resolution occurs in favor of either BAS mediated approach (perception of danger has diminished) or FFFS-mediated active avoidance or escape (perception of danger is now more apparent and/or increased).
	Trait-FFFS	Corr and Cooper (2016, p. 1428): “… a punishment sensitivity system responsible for mediating reactions to unconditioned aversive, pain-inducing stimuli only.”
	Trait-BAS	Corr and Cooper (2016, p. 1429): “… some form of “subgoal scaffolding” is required (Corr, 2008). This process consists of (a) identifying the biological reinforcer, (b) planning behavior, and (c) executing the plan …”
revised Reinforcement Sensitivity Theory-Questionnaire (rRST-Q)	Trait-BAS	Reuter et al. (2015, p. 2): “… characterized as full of energy, having a tendency toward outgoing explorative behavior, and being more motivated to pursue rewards.”
	Trait-BIS	Reuter et al. (2015, p. 2): “…attributed to goal conflict. …BIS activation is thought to include careful and slow approach behavior toward the potentially dangerous stimuli and risk assessment behavior.”
	Trait-FFFS	Reuter et al. (2015, p. 7): “reflecting the emotion of fear”; “…measuring individual differences in fear tendencies, comprises the most important classes of behavioral fear responses, namely Fight, Flight, and Freezing”

The factorial validity of the German Carver-White BIS/BAS scales, the RST-PQ, and the rRST-Q has been confirmed by means of CFAs (Strobel et al., 2001; Reuter et al., 2015; Pugnaghi et al., 2018). Further studies have examined alternative models of the Carver-White BIS/BAS scales—-especially a two-factor model of the trait-BIS scale in addition to the three trait-BAS subscales (Johnson et al., 2003; Heym et al., 2008; Levinson et al., 2011; Müller et al., 2013; Maack and Ebesutani, 2018). In addition to CFA studies for the Carver-White BIS/BAS scales, further CFA models were tested for the RST-PQ (Krupić et al., 2016; Wytykowska et al., 2017; Eriksson et al., 2019). For a short RST-PQ scale see Veccione and Corr (2020).

As a new development, the conflict monitoring questionnaire (CMQ, Leue and Beauducel, 2021) investigates determinants of revised trait-BIS-related conflict monitoring by means of anticipation of negative consequences (Gray and McNaughton, 2000; Corr, 2008) and cognitive demand (Botvinick, 2007; Leue et al., 2012, 2014). Response adaptation and uncertainty of reinforcement are assessed as behavioral consequences following stimulus-related conflict monitoring (named as “response patterns following conflict monitoring” in Leue and Beauducel, 2021, Table 1). The CMQ-44 (i.e., “44” deriving from the fact that 44 of 60 items with the best psychometric properties are analyzed, see “Materials and Methods” section) has been developed based on facet theory (Shye et al., 1994; Guttman and Greenbaum, 1998; Hackett, 2014) meaning that each item contains a determinant and a consequence of conflict monitoring (Leue and Beauducel, 2021). In sum, the present study aims at investigating construct validity (Cronbach and Meehl, 1955; Campbell and Fiske, 1959) of the (r)RST-related questionnaires within one sample allowing also to compare pre-processing issues of the items (see section Parceling issues; research question 2).

### Measurement equivalence

Effects of measurement equivalence have been rarely addressed in previous CFA studies on (r)RST questionnaires. Beyond CFA modeling, effects of gender and/or age groups have been reported on the scale-level for the German version of the Carver-White BIS/BAS scales (Strobel et al., 2001) and the rRST-Q of Reuter et al. (2015). Correspondingly, trait-BIS and trait-FFFS (Flight and Freeze) mean values were slightly higher in female compared to male participants, but not for trait-BAS (Reuter et al., 2015, their Table 3). Trait-BIS and trait-BAS were significantly higher for females than males (Strobel et al., 2001). Age group effects were not significant (Strobel et al., 2001). Leue and Beauducel (2021) also reported gender effects meaning that women reported higher CMQ-44 cognitive demand and CMQ-44 performance monitoring than men. Therefore, this study investigates a Multiple-Indicator-Multiple-Cause (MIMIC) model for the Carver-White BIS/BAS scales, the RST-PQ, the rRST-Q, and for the CMQ-44. A MIMIC model has been preferred over other statistical models (e.g., multiple group confirmatory factor analysis) in purpose of comparison with the original factorial validation of the CMQ-44 published in Leue and Beauducel (2021). We address gender as a MIMIC factor in all CFA models (research question 3).

Additionally, none of the (r)RST CFA studies investigated a hierarchical structure of the BIS/BAS sub-scales. As Leue and Beauducel (2021) indicated, the CMQ-44 allows for a hierarchical factor model including performance monitoring (G) as a second-order factor and four first-order factors (cognitive demand, anticipation of negative consequences, response adaptation, uncertainty of reinforcement). Performance monitoring has been correlated with the other (r)RST first-order factor scores (Results section) to evaluate the generality of the first-order factors in non-hierarchical (r)RST models.

### Previous results on convergent and discriminant validity among (r)RST trait-scales

Previous studies revealed positive inter-correlations between trait-BIS scales of the Carver-White BIS/BAS scales (Strobel et al., 2001), the RST-PQ (Pugnaghi et al., 2018), and the rRST-Q (Reuter et al., 2015). Similarly, positive inter-correlations have been reported between trait-BAS scales of the Carver-White BIS/BAS scales (Strobel et al., 2001), the RST-PQ (Pugnaghi et al., 2018), and the rRST-Q (Reuter et al., 2015). Inter-correlations between trait-BIS and trait-BAS scales were often significantly negative or non-significant. Therefore, we presume evidence of convergent validity among trait-BIS and among trait-BAS scales, respectively. In contrast, we presume evidence of discriminant validity between trait-BIS and trait-BAS scales.

Cognitive demand has been discussed in the context of the conflict-monitoring-theory (Botvinick, 2007) as a determinant that enhances conflict monitoring. Cognitive demand of the CMQ-44 measures the tendency to respond to situational or experimental requirements of higher cognitive demand by an intensification of conflict monitoring, performance monitoring and subsequently cognitive control (Leue et al., 2012, 2014). In this line, we predict that cognitive demand of the CMQ-44 correlates positively with other personality scales of (r)RST questionnaires that aim to measure trait-BIS (i.e., the tendency to detect and control for conflict information). As self-reports of higher cognitive demand should be related to cautious behavior, we predict negative correlations with behavioral approach tendencies measured with the trait-BAS scales. According to rRST (Gray and McNaughton, 2000; Corr, 2008), individuals with higher trait-BIS scores anticipate negative consequences of errors and, therefore, invest in a more intense stimulus monitoring compared to individuals with lower trait-BIS scores and individuals with higher compared to lower reasoning ability (Leue et al., 2014). Therefore, we presume that the CMQ-44 anticipation of negative consequences is positively correlated with (r)RST trait-BIS scales. In contrast, we predict no substantial or negative correlations with (r)RST trait-BAS scales because we expect the anticipation of negative consequences scale to be rather related to stimulus monitoring and evaluation than to reward-related approach behavior as measured by means of trait-BAS scales.

The CMQ-44 response adaptation scale is thought to be positively related to previous (r)RST-related trait-BIS scales because CMQ-44 response adaptation can serve to inhibit behavior especially when response adaptation occurs in a less adaptive manner. When CMQ-44 response adaptation is performed in a reactive, more flexible manner, higher scores of the CMQ-44 response adaptation scale could also correlate positively with trait-BAS scales and negatively with previous trait-(r)BIS scales. If the CMQ-44 response adaptation correlates negatively with previous trait-(r)BIS scales this would indicate that response adaptation extends previous trait-BIS scales by measuring a more flexible, reactive adaptation of responses instead of a fixed, proactive adaptation of responses (Braver, 2012).

CMQ-44 uncertainty of reinforcement measures the tendency to be sensitive for situations that are ambiguously reinforcing because situations are rewarding as well as punishing or they are so complex for the decision process that the reinforcing value cannot be defined. CMQ-44 uncertainty of reinforcement should enhance behavioral inhibition tendencies and, therefore, correlates positively with (r)RST-related trait-BIS scales. Moreover, higher scores of CMQ-44 uncertainty of reinforcement should reduce behavioral approach tendencies and, therefore, -if at all- is negatively related to (r)RST trait-BAS scales. Presuming that people take the risk of errors into account, higher scores of CMQ-44 uncertainty of reinforcement could be positively related to approach behavior as measured with trait-BAS scales. The predictions of the CMQ-44 subscales with previous (r)RST-related trait-BIS and trait-BAS scales are summarized in Table 2 and address research question 4.

**TABLE 2:** Summary of predictions for CMQ-44 subscales and (r)RST-questionnaires.

	CMQ-44-determinants		CMQ-44-consequences	
	Cognitive demand	Anticipation of negative consequences	Response adaptation	Uncertainty of reinforcement
(r)RST Trait-BIS scales	+	+	±	+
(r)RST Trait-BAS scales	–	–/ns	–/ns	±

“–”, negative significant correlation; “ + “, positive significant correlation. “± “, correlation can be predicted based on literature to be negative or positive and significant. “–/ns”, direction of correlation is predicted to be negative and significant or non-significant. No *a priori* predictions were performed for correlations between Trait-BIS and Trait-BAS scales with Trait-FFFS scales of the rRST-Q and the RST-PQ, respectively. Following Campbell and Fiske (1959, p. 84) evidence of discriminant validity can result in negative correlations.

### Parceling issues in (r)RST questionnaires

Factorial validity of the (r)RST questionnaires – except the CMQ-44 – have been mainly investigated on the item-level. Parceling items (Humphreys, 1962) has been mainly applied for items constructed based on facet theory (Liepmann et al., 2007; Beauducel and Kersting, 2010; Beauducel et al., 2010; Leue and Beauducel, 2021). Parceling items allows us to capture systematic item content prior to the investigation of factorial validity by means of CFA models and prior to the calculation of unit-weighted sum scales or factor scores. Due to a rational, theory-driven item construction, researchers define *a priori* which items are thought to measure the latent constructs or at least parts of those constructs (Süß and Beauducel, 2005). In this respect, parceling items has the advantage that items can be systematized based on their *a priori* defined item content and tested for their model fit with the conceptually intended scales. Beyond these advantages, item parceling has been an issue of psychometric critics (Marsh et al., 2013). Marsh et al. (2013) argue that parceling items is “never appropriate *a priori*” (p. 258) because item misspecifications are ignored otherwise. The argument probably neglects the fact that items for the measurement of personality traits and intelligence have been sometimes constructed in an inductive, empirical manner rather than in a theory-driven approach as recommended by means of facet theory (Guttman and Greenbaum, 1998). Thus, item parceling for items constructed based on theoretical predictions as (r)RST and facet theory particularly summarizes those items that have been conceptualized *a priori* to belong to a certain construct or a sub-facet of a construct. In this respect, item parceling prior to the investigation of the model fit in CFA models can be conceived as a necessary pre-processing step – not as a prevention of item misspecifications. Moreover, results of CFA models indicate that –despite item parceling– not all theory-driven CFA models show a sufficient or very good model fit (see Results section). To investigate construct validity in a test-fair manner for all (r)RST questionnaires in this study, items of all (r)RST questionnaires have been parceled with regard to their *a priori* defined construct content (Sterba and Rights, 2017). This procedure ensures that theory-driven item development and item development based on facet theory can be tested for comparable and test-fair item units. If item parceling would not be applied in a comparable way to all (r)RST questionnaires, construct validity of the (r)RST items would have been compared on different construct levels. Performing the CFA models for all (r)RST questionnaires allows us to save factor scores of all latent factors. The factor scores for the Carver-White BIS/BAS scales, the RST-PQ, and the rRST-Q were applied to investigate the inter-correlations with the factor scores of the CMQ-44 subscales. Otherwise, inter-correlations that would have been calculated for unit-weighted sum scales in some (r)RST questionnaire and factor scores in the CMQ-44 might underestimate or overestimate inter-correlations because of scaling issues.

### Aims and research questions

Based on prior findings we investigate the following research questions. (1) Are psychometric properties for the (r)RST-related questionnaires comparable to prior findings? (2) Can factorial validity of the four German (r)RST questionnaires (i.e., Carver-White BIS/BAS scales, RST-PQ, RST-Q, and CMQ-44) be confirmed based on the parcel level? (3) Are (r)RST latent factors equivalent across gender? (4) Do the factor scores for the best fitting CFA models provide evidence of *a priori* predicted convergent and discriminant validity (Table 2)?

## Materials and methods

### Sample

A total of *N* = 1,127 participants took part in a Unipark survey that was performed in collaboration with Respondi AG^1^ between November 2020 and January 2021 (*n* = 88 were assessed via Unipark by a research assistant in the team of the first author). The psychometric survey along with the research questions was approved in September 2020 by the Ethics committee of the Medical Faculty at the University of Kiel, Germany. Hypotheses in Table 2 were not pre-registered but formulated *a priori* (i.e., prior to data collection). We recruited participants in three examination intervals via Respondi AG. The first examination was performed between 24-November-2020 and 13-December-2020 with *n* = 44 participants in a pre-test and *n* = 471 participants in the main test. The second examination interval included *n* = 2 participants in the pre-test and *n* = 523 in the main test lasting from 13-January-2021 until 25-January-2021. The third Unipark assessment started on 17-December-2020 and ended on 20-February-2021 with *n* = 5 participants in a pre-test and *n* = 83 participants who took part in the main test (*n* = 88 see above). Overall, of the *N* = 1,128 participants we excluded *n* = 51 pre-test participants because pre-tests included slight changes in the Unipark programming. One participant younger than 18 years was excluded.

The final sample comprised *N* = 1,076 participants aged between 18 and 66 years (*M* = 38.38 years, *SD* = 12.93) for statistical analysis. We planned a widely equal recruitment of four age groups between 18 and 28 years (*n* = 317), 29 and 39 years (*n* = 250), 40 and 50 years (*n* = 286), and 51 and 66 years (*n* = 223). A total of *n* = 514 female and *n* = 559 male participants took part in this study (for gender proportions in Germany^2^). Three participants classified their gender as diverse. Participants received a reimbursement credit via Respondi AG redirects of about 5 €. The study plan was pre-registered in Hogrefe Verlag, Germany (proposal sent in April 2020 and discussed with a member of the Hogrefe Verlag in the beginning of May 2020). Data acquisition was funded by the University of Kiel, Germany.

### Inventories

Participants were asked to answer demographic variables (e.g., federal state, age, gender, school grade, profession, income per month). Afterward participants answered the items of four questionnaires in German language in the following sequence: (1) Conflict monitoring questionnaire CMQ-44 (Leue and Beauducel, 2021), (2) Reinforcement Sensitivity Theory – Personality Questionnaire (RST-PQ, Pugnaghi et al., 2018), (3) BIS/BAS scales (Strobel et al., 2001), and (4) Reuter-Montag’s rRST questionnaire (rRST-Q, Reuter et al., 2015). For item examples the publications cited in (1) to (4) should be consulted.

The CMQ originally includes 60 items with a 6-point Likert response scale: 1 = trifft überhaupt nicht zu (does not correspond at all), 2 = trifft überwiegend nicht zu (does mainly not correspond), 3 = trifft eher nicht zu (does rather not correspond), 4 = trifft eher zu (does rather correspond), 5 = trifft überwiegend zu (does mainly correspond), 6 = trifft vollständig zu (does completely correspond). The CMQ incorporates four latent factors named as structs in terms of facet theory (Table 1). Two latent factors describe determinants of conflict monitoring: cognitive demand and anticipation of negative consequences. Two further latent factors differentiate behavioral consequences of conflict monitoring and are entitled as response adaptation and uncertainty of reinforcement. Higher self-reported cognitive demand and anticipation of negative consequences are thought to be related to more intense conflict monitoring. Higher self-reported response adaptation and experience of uncertainty of reinforcement are thought to result from more intense conflict monitoring (for factor meanings see Leue and Beauducel, 2021, section 3.3 “Quality assessment”). Of the 60 items, the shorter versions CMQ-44 and CMQ-28 comprise Cronbach’s Alpha coefficients between 0.72 and 0.89 and revealed sufficient to very good psychometric properties (Leue and Beauducel, 2021, their Table 6).

The German version of the RST-PQ (Pugnaghi et al., 2018) includes 65 items with four response categories of a 4-point Likert type with 1 = überhaupt nicht (not at all), 2 = etwas (slightly), 3 = mäßig (moderately), 4 = sehr (highly) but varying verbal coding compared to Strobel et al. (2001) and Reuter et al. (2015). The BAS scale differentiates four subscales entitled BAS – reward interest, BAS – goal drive persistence, BAS – reward reactivity, BAS – impulsivity. The BIS scale incorporates four subscales named as BIS – cautious risk assessment, BIS – motor planning interruption, BIS – behavioral disengagement, and BIS – obsessive thoughts. The FFFS scale includes Flight, Active Avoidance, and Freezing. All personality scales and sub-scales revealed a Cronbach’s Alpha between 0.67 and 0.91 (Pugnaghi et al., 2018, their Table 1). The BAS subscales in Strobel et al. (2001) are entitled reward responsiveness, fun seeking and drive. Thus, whereas Reuter et al. (2015) and Pugnaghi et al. (2018) disentangled the FFFS subscales, Strobel et al. (2001) and Pugnaghi et al. (2018) differentiated the BAS subscales. The German BIS/BAS scales as a translation of the English BIS/BAS scales (Carver and White, 1994) consist of 24 items (four dummy items are not included into statistical analysis). Cronbach’s Alpha has been reported for the Carver-White BIS/BAS scales with 0.67–0.81 (Strobel et al., 2001, Table 3). The rRST-Q incorporates 31 items with a Cronbach’s Alpha reliability ranging between 0.75 and 0.78 (Reuter et al., 2015, Table 4). The rRST-Q measures trait-BIS, trait-BAS and trait-FFFS (including Fight, Flight and Freezing behavior). Both the BIS/BAS scales and the rRST-Q apply a 4-point Likert-type response format with 1 = trifft für mich gar nicht zu (I strongly disagree), 2 = trifft für mich eher nicht zu (I disagree), 3 = trifft für mich eher zu (I rather agree), and 4 = trifft für mich genau zu (I strongly agree).

### Procedure

At the start of the Unipark link, participants were informed about the study, duration per examination (about 30–40 min), and contact persons who were prepared to answer questions on the study. Participants were instructed to answer the items in a well-lit, quiet room with no disturbance during item answering and no participation of others. When participants gave written informed consent, they obtained demographic and questionnaire items for answering. Respondi AG handled the recruitment and reimbursement (mingle points which could be converted in the Respondi AG portal) of most participants (except *n* = 88 who were recruited and reimbursed at the University of Kiel, Germany, in the team of the first author).

### Statistical analysis

Statistical analysis was performed using IBM SPSS statistics version 26 and Mplus version 8.3 (Muthén and Muthén, (1998–2017)). Preprocessing of data included the investigation of missing values and normal distribution. There were no missing values because participants answered all items. By using SPSS 26, we performed Mardia’s test of multivariate kurtosis to test for multivariate normal distribution (DeCarlo, 1997). The Mardia’s test was performed on parcel level. Parcels were performed based on an *a priori* questionnaire construction, i.e., items belonging to the same item content were grouped into parcels (Little et al., 2002). That means by reading the published items parceling was performed. We performed sum scores to establish the item parcels. Supplementary Table 1 provides an overview of the items per parcel. Each item was applied once for computing a parcel to hold the criterion of a theory-related item-to-parcel allocation (Sterba and Rights, 2017). Mardia’s test was significant for all (r)RST questionnaires included in this study (Supplementary Table 1) suggesting that the multivariate normal distribution was not given. Therefore, we applied a maximum likelihood estimator with robust standard errors entitled as MLR in our CFA models (Luo, 2018). Mardia’s test was preferred over Q–Q plots because Mardia’s test allows for a statistical instead of a graphical evaluation of multivariate normal distribution.

We report model fit (Hu and Bentler, 1999; Beauducel and Wittmann, 2005) for the following indices: Comparative Fit Index (CFI), Root Mean Square Error of Approximation (RMSEA), Standardized Root Mean Square Residual (SRMR). Results showing a CFI of better than 0.90 (Beauducel and Wittmann, 2005) and 0.95 (Hu and Bentler, 1999) were evaluated as very good. We decided to evaluate a range for the CFI because the thresholds for the description of the CFI differ with regard to software, tested factor loading thresholds and number of factors in a CFA model (Hu and Bentler, 1999; Beauducel and Wittmann, 2005). A RMSEA of ≤0.06 and a SRMR of ≤0.04 (Beauducel and Wittmann, 2005, their Table 3) was evaluated as a very good model fit. Construct validation in this study incorporates the investigation of the factorial validity of the four questionnaires on parcel-level by means of CFA and inter-correlations of factor scores. We report factor loadings and MIMIC findings for gender of the STDYX matrix. Spearman Rank correlations were calculated to report findings on convergent and discriminant validity for the factor scores. Partial correlations are reported to indicate effects of gender on the convergent and discriminant results. As all inventories are part of an on-going construct validation process original data, code books, or program code will be made available in PsyArxiv upon request to the first author and depending on further validation studies: https://osf.io/9vu8e/?view_only=9655b511443c4c5e95f9393fcb15622c.

## Results

### Psychometric and descriptive data (research question 1)

In a sample of *N* = 1,076 participants we observed the following psychometric properties for trait-BIS and trait-BAS scales, and FFFS scales (Table 3). Excellent reliabilities (≥0.90, given in bold in Table 3) were rare for the (r)RST questionnaires (Table 3 and Supplementary Table 2). Most reliabilities were moderate (0.80–0.90) and are given in italics in accordance with George and Mallery (2020). Reliabilities were comparable or even higher in the present study (Table 3 and Supplementary Table 2) compared to previous studies (Strobel et al., 2001; Reuter et al., 2015; Pugnaghi et al., 2018; Leue and Beauducel, 2021). Thus, reliabilities suggest moderate to high data quality for the present online data. Supplementary Table 2 summarizes descriptive statistics of all (r)RST questionnaires.

**TABLE 3:** Psychometric properties of the (r)RST subscales (*N* = 1,076).

(r)RST scale	No. items	Range of item means	Range of corrected item-total correlations: based on pearson correlations	Range of corrected item-total correlations: based on Spearman’s rho	Hancock’s *H* based on regression factor scores
CW-BIS	7	2.53–3.11	0.46–0.69	0.47–0.67	0.88/0.88^1^
CW-BAS	13	2.58–3.18	0.31–0.61	0.28–0.60	*0.85*
CW-BAS-FS	4	2.58–3.13	0.37–0.56	0.34–0.56	0.77
CW-BAS-RR	5	2.92–3.42	0.36–0.59	0.36–0.60	0.70
CW-BAS-Drive	4	2.77–3.05	0.46–0.63	0.47–0.64	*0.85*
RST-PQ-BIS: MPI	5	2.08–2.67	0.43–0.60	0.42–0.58	**0.91**
RST-PQ-BIS: CRA	5	2.35–2.62	0.53–0.71	0.52–0.71	**0.94**
RST-PQ-BIS: OT	7	2.18–2.87	0.62–0.80	0.61–0.79	**0.95**
RST-PQ-BIS: BD	6	2.06–2.67	0.55–0.72	0.56–0.71	**0.93**
RST-PQ-BAS: RI	7	2.21–2.98	0.50–0.65	0.49–0.64	*0.86*
RST-PQ-BAS: GDP	7	2.74–3.12	0.45–0.70	0.46–0.69	*0.89*
RST-PQ-BAS: RR	10	2.26–3.39	0.33–0.63	0.36–0.61	*0.84*
RST-PQ-BAS: Imp	8	1.78–2.97	0.32–0.57	0.31–0.56	*0.80*
RST-PQ-BIS	23	2.06–2.87	0.42–0.77	0.41–0.80	**0.94**
RST-PQ-BAS	32	1.78–3.39	0.22–0.62	0.20–0.56	**0.90**
RST-PQ-FFFS	10	1.96–2.71	0.28–0.59	0.28–0.59	*0.80*
rRST-Q: BIS	11	1.97–2.90	0.25–0.69	0.28–0.65	*0.87/0.86^+^*
rRST-Q: BAS	8	2.25–2.90	0.46–0.60	0.43–0.58	*0.87/0.82^+^*
rRST-Q: FFFS	11^#^	2.18–2.61	0.24–0.61	0.23–0.62	*0.81^+^*
CMQ-44-CD	7	2.97–3.77	0.31–0.57	0.28–0.54	0.78^a^
CMQ-44-ANC	7	3.26–4.13	0.30–0.64	0.34–0.66	0.66^a^
CMQ-44-RA	16	3.15–4.25	0.13–0.74	0.17–0.72	*0.84^a^*
CMQ-44-UR	14	3.38–4.16	0.10–0.71	0.09*–0.69	*0.85^a^*
CMQ-28-CD	7	2.97–3.77	0.31–0.57	0.28–0.54	0.65^a^
CMQ-28-ANC	7	3.26–4.13	0.33–0.67	0.34–0.66	0.74^a^
CMQ-28-RA	7	3.41–3.74	0.61–0.75	0.60–0.73	0.79^a^
CMQ-28-UR	7	3.38–4.16	0.60–0.68	0.57–0.66	0.73^a^

The 7 items of the cognitive demand (CD), the ANC (Anticipation of negative consequences), the RA (Response adaptation) and the UR (Uncertainty of reinforcement) structs form the CMQ-28 (Leue and Beauducel, 2021). *Item 56 of the UR struct. #As item 27 had a negative part-whole corrected item-total correlation, it was excluded from further statistical analysis.

^1^Hancock’s *H* of the Carver-White (CW) trait-BIS scale of the four-factor model including CW trait-BIS and three CW trait-BAS subscales. ^+^Hancock’s *H* of the rRST-Q trait-BIS, trait-BAS, and trait-FFFS scale of the three-factor model. Hancock’s *H* includes factor loadings of the STDYX matrix, which was calculated based on parcels as reported in Supplementary Table 1 for Mardia’s test of normality. ^a^For the CMQ-44 and the CMQ-28, we report the factor determinacy reliabilities presented in Mplus, which are comparable estimates to Hancock’s *H*. The factor determinacy reliabilities for the CMQ-44 and for the CMQ-28 are based on the parcels given in Supplementary Table 1. Whereas internal consistency coefficients are identical for the seven items of Cognitive demand (CD) and for the seven items of Anticipation of negative consequences (ANC) for the CMQ-44 and the CMQ-28 (Supplementary Table 3), the CMQ-28 factor determinacy reliabilities differ because the factor loadings in both CMQ versions differ.

### Factorial validity of (r)RST trait-BIS-related, trait-FFFS-related and trait-BAS-related subscales (research question 2)

Table 4 summarizes all performed CFA MIMIC models. Item parcels were performed based on the ascending item number for each latent, theory-driven content factor (i.e., for BIS and BAS items, see Supplementary Table 2) of the RST-PQ, the rRST-Q, and the Carver-White BIS/BAS scales. Briefly, our results show that a very good model fit occurred for the four-factors Carver-White-BIS/BAS model and for the 4-factors RST-PQ model in terms of CFI and RMSEA (Table 4, model fit indices marked in bold). For the two-factors rRST-Q, and for the CMQ-44 or 28 bifactor models the model fit was very good in terms of CFI, RMSEA and SRMR (Table 4, model fit indices marked in bold).

**TABLE 4:** Results of the CFA MIMIC models (*N* = 1,076).

	χ^2^-test	CFI	RMSEA	SRMR
**CW-BIS/BAS**				
Model 1: 2-factors	63.16** (*df* = 8)	**0.97**	0.08	0.09
Model 2: 4-factors	95.34** (*df* = 21)	**0.98**	**0.06**	0.08
**RST-PQ**				
Model 1: 2-factors	728.43** (*df* = 89)	**0.93**	0.08	0.08
Model 2: 3-factors-BIS, BAS, FFFS	995.31** (*df* = 150)	**0.92**	0.08	0.08
Model 3: 4-BIS/4-BAS factors	556.61** (*df* = 106)	**0.96**	**0.06**	0.08
**rRST-Q**				
Model 1: 2-factors	41.17** (*df* = 8)	**0.98**	**0.06**	**0.03**
Model 2: 3-factors	492.49** (*df* = 25)	0.88	0.13	0.14
**CMQ-44**				
Model 1: 4-factors	2008.97** (*df* = 148)	0.82	0.11	0.28
Model 2: bifactor with 4-factors	601.92** (*df* = 129)	**0.95**	**0.06**	**0.04**
**CMQ-28**				
Model 1: 4-factors	1759.97** (*df* = 51)	0.74	0.18	0.29
Model 2: bifactor with 4-factors	355.90** (*df* = 38)	**0.95**	**0.09**	**0.03**

For the CMQ-44, model 2 the parcel RA_p1 was fixed to one because otherwise the factor loading was larger than 1. **p < 0.01.

All three two-factor models of the Carver-White BIS/BAS scales, the RST-PQ, and of the rRST-Q are primary-order factor models (Table 4). The two-factors Carver-White trait-BIS/trait-BAS model including two trait-BIS parcels with three to four items per parcel and three trait-BAS parcels with four to five items per parcel (Supplementary Table 1) showed a very good model fit for the CFI and a moderate model fit for the RMSEA and SRMR. In accordance with Strobel et al. (2001), the inter-correlation between trait-BIS and trait-BAS was set to 0.17 to perform the MIMIC models (Table 4).

The two-factor RST-PQ model for trait-BIS and trait-BAS including parcels of three to four items (Supplementary Table 1) suggest again a very good model fit for the CFI and a moderate model fit for the RMSEA and the SRMR (Table 4). In accordance with Pugnaghi et al. (2018, their Table 1), prior to the calculation of the MIMIC model we set the inter-correlation between trait-BIS and trait-BAS to –0.02 which corresponds to the mean inter-correlations between trait-BIS and the trait-BAS subscales. For the three-factor model of the RST-PQ the model fit was poor. The mean inter-correlation between trait-BAS and trait-FFFS was 0.08. Trait-BIS and trait-FFFS were set to an inter-correlation of 0.46 (see Pugnaghi et al., 2018, their Table 1).

**FIGURE 1:**
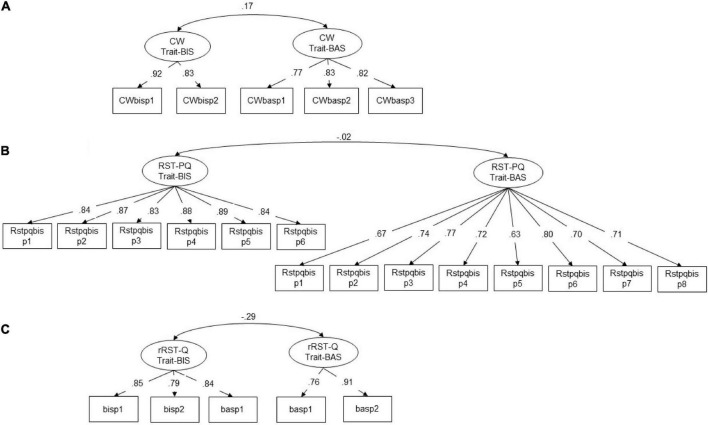
Standardized factor loadings (STDYX) of two-factor models for the Carver-White (CW) BIS/BAS questionnaire **(A)**, RST-PQ **(B)**, and Reuter-Montag (RM) rRST-Q **(C)**. p1–p8, parcel 1–parcel 8. All *p*-values *p* < 0.01, two-tailed.

The two-factor rRST-Q trait-BIS and trait-BAS model showed a very good model fit in terms of CFI, RMSEA, and SRMR based on parcel-level (Table 4) with three to four items per parcel (Supplementary Table 1). The model fit of the three-factor model including trait-BIS, trait-BAS, and trait-FFFS was poor (Table 4). In accordance with Reuter et al. (2015, their Table 6) we set the inter-correlation between trait-BIS and trait-BAS to –0.29, the inter-correlations between trait-BIS and trait-FFFS to 0.45 and to –0.41 for trait-BAS and trait-FFFS prior to the calculation of the MIMIC model. Figure 1 summarizes the standardized factor loadings (STDYX) on parcel level for the two-factor models of the Carver-White BIS/BAS questionnaire, the RST-PQ and the rRST-Q.

**FIGURE 2:**
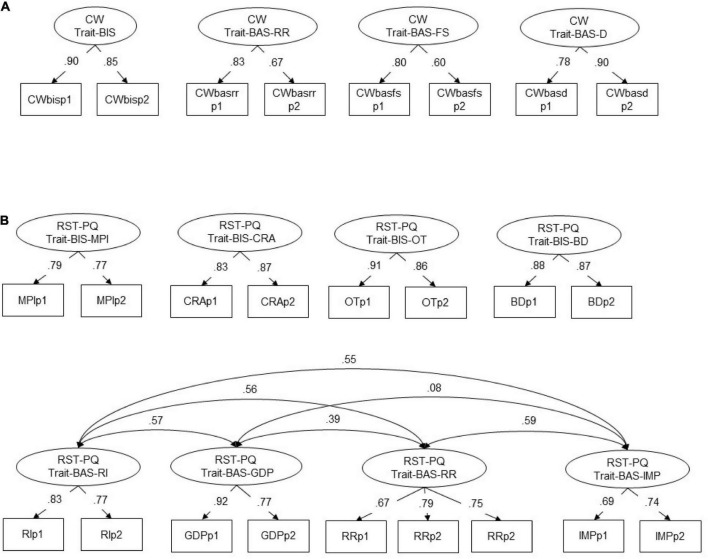
Standardized factor loadings (STDYX) of four-factor models for the Carver-White BIS/BAS questionnaire including one BIS and three BAS subfactors **(A)** and for the RST_PQ including four BIS and four BAS subfactors **(B)**. BD, behavioral disengagement; CRA, cautious risk assessment; D, drive; GDP, goal-drive persistence; FS, fun seeking; IMP, impulsivity; MPI, motor planning interruption; OT, obsessive thoughts; RI, reward interest; RR, reward reactivity; CW-trait-BAS-RR, reward responsiveness. All *p*-values *p* < 0.01, two-tailed.

As trait-BIS and trait-BAS subfactors have been reported for the Carver-White BIS/BAS questionnaire and for the RST-PQ, we additionally performed primary factor MIMIC CFAs for more than two latent factors (Table 4 and Figure 2A). For the Carver-White BIS/BAS questionnaire, we performed a model including trait-BIS and three latent factors entitled trait-BAS – reward responsiveness, trait-BAS – fun seeking, and trait-BAS – drive. Item parcels were performed based on item content (i.e., items that were thought to belong to the respective sub-scale). Item parcels for the trait-BAS subscales incorporate two to three items of a conceptually comparable content per parcel. Each BAS subfactor comprised two parcels. The four-factor model with trait-BIS and three trait-BAS subscales of the Carver-White BIS/BAS scales fitted the data well in terms of CFI and RMSEA (Table 4).

For the RST-PQ we performed a 4-factor model including four trait-BIS subfactors and four trait-BAS subfactors (Table 4 and Figure 2B). The trait-BIS subscales are entitled as Motor planning interruption, Cautious risk assessment, Obsessive thoughts, and Behavioral disengagement. Item parcels of the RST-PQ trait-BIS subfactors incorporate two to four items per parcel. The trait-BAS subfactors for the RST-PQ are named as Reward interest, Goal-drive persistence, Reward reactivity, and Impulsivity. Again, item parcels of the RST-PQ trait-BAS subfactors comprise three to four items per parcel. The four-subfactors model for trait-BIS and the four-subfactors model for trait-BAS fitted the data very well in terms of CFI and RMSEA (Table 4). The inter-correlations between the four trait-BAS factors were chosen as reported in Pugnaghi et al. (2018). No inter-correlations were reported for the four trait-BAS factors in Pugnaghi et al. (2018).

Regarding the model fit of the CMQ-44, we performed a four factors primary-order MIMIC model including cognitive demand, anticipation of negative consequences, response adaptation, and uncertainty of reinforcement. The model did not fit the data well (Table 4). In contrast, a bifactor MIMIC factor model of the CMQ-44 showed a very good model fit (Table 4) as recently reported in Leue and Beauducel (2021). The standardized factor loadings for the bifactor MIMIC factor model were – except a few loadings – significant (Table 5 and Supplementary Figure 1). For CMQ-28 (Supplementary Figure 2 and Supplementary Table 4), the model fit results were pretty comparable to the CMQ-44 for the CFI, RMSEA, and SRMR. For the CMQ-44 and for the CMQ-28 the latent factors were presumed to be orthogonal (i.e., no factor inter-correlations were specified for the MIMIC models).

**TABLE 5:** Standardized factor loadings (STDYX) of the Bifactor MIMIC model of the CMQ-44 (*N* = 1,076).

Parcel	Factor loadings
	First-order trait factors
**Cognitive demand**	
DU_p1 (14d_r, 19)	0.07
DU_p2 (24, 52d_r)	0.15***
DR_p3 (47, 49d_r, 58)	–0.08(*)
UD_p2 (08u_r, 46)	0.13***
UD_p3 (37, 16u_r)	0.13**
UD_p4 (41, 27u_r)	0.23***
UD_p5 (50, 56)	–0.10*
RD_p3 (01, 53)	0.35***
RD_p4 (17r_r, 22)	0.31***
RD_p5 (06, 30)	0.28***
RD_p6 (31, 35)	0.38***
RD_p7 (38, 43, 25)	0.38***
**Anticipation of negative consequences**	
AU_p1 (10a_r, 20a_r, 23)	–0.19**
AU_p2 (36a_r, 59a_r)	0.04
AR_p3 (44a_r, 54a_r)	0.19**
RA_p1 (02, 21r_r, 57r_r)	0.25***
RA_p2 (48, 39)	–0.17***
UA_p1 (04u_r, 07, 12u_r)	0.27**
UA_p6 (40, 45, 55u_r)	–0.04
**Response adaptation**	
RA_p1 (02, 21r_r, 57r_r)	0.70***
RA_p2 (48, 39)	0.07(*)
RD_p3 (01, 53)	0.14***
RD_p4 (17r_r, 22)	0.39***
RD_p5 (06, 30)	0.08*
RD_p6 (31, 35)	0.08*
RD_p7 (38, 43, 25)	0.04
DR_p3 (47, 49d_r, 58)	–0.30***
AR_p3 (44a_r, 54a_r)	–0.05
**Uncertainty of reinforcement**	
UA_p1 (04u_r, 07, 12u_r)	0.38***
UD_p2 (08d_r, 46)	0.52***
UD_p3 (37, 16u_r)	0.40***
UD_p4 (41, 27u_r)	0.46***
UD_p5 (50, 56)	–0.21***
UA_p6 (40, 45, 55u_r)	0.37***
DU_p1 (14d_r, 19)	0.26***
DU_p2 (24, 52d_r)	–0.10(*)
AU_p1 (10a_r, 20a_r, 23)	–0.11*
AU_p2 (36a_r, 59a_r)	0.34***

Each parcel contains the items reported in the Table and has been computed as a sum score. (*)*p* < 0.10, *p ≤ 0.05, **p < 0.01, and ***p < 0.001 (all *p*-values two-tailed). An item has been indicated with “_r” when the first part of the item incorporated a struct that was not coded in the direction of high cognitive demand (D), high anticipation of negative consequences (A), high response adaptation (R), or high uncertainty of reinforcement (U). That is a primacy effect of item reading and processing was the basis for recoding an item (cf. Leue and Beauducel, 2021). Standardized factor loadings (STDYX) of the second-order factor G: performance monitoring are given in Supplementary Figure 1.

### Measurement equivalence across gender (research question 3)

Effects of measurement equivalence across gender have not been predicted *a priori* as directed hypotheses in this study. This is due to the fact that gender effects in Strobel et al. (2001), Reuter et al. (2015), and Leue and Beauducel (2021) (section “Characteristics of included studies”) were calculated based on quite different statistical methods (ANOVA, MIMIC models). Significant gender differences were observed for the trait-BIS factor (β = 0.36, *p* < 0.01) and for trait-BAS in the Carver-White BIS/BAS two factor model (β = 0.18, *p* < 0.01) with women (*n* = 514) and individuals who classified themselves as diverse (*n* = 3) showing higher trait-BIS and higher trait-BAS values than men (*n* = 559). In the Carver-White BIS/BAS four factor model, female and diverse individuals showed higher trait-BIS, trait-BAS-reward reactivity and trait-BAS-drive values than men (for all three latent factors: β = 0.36, 0.13, 0.07, *p*s < 0.05).

For the RST-PQ two-factor model, women revealed higher trait-BIS values (β = 0.20, *p* < 0.01) and higher trait-BAS values (β = 0.07, *p* < 0.05) than men. Regarding the RST-PQ four-factor models for trait-BIS and trait-BAS, respectively, women and individuals who classified themselves as diverse scored higher than men for all four trait-BIS subscales (β = 0.17 to 0.25, *p*s < 0.001). For the trait-BAS subfactors exclusively Goal-drive persistence and Reward reactivity were higher for female and diverse participants compared to men (both latent factors: β = 0.10, *p*s < 0.01).

For the rRST-Q, women and individuals who classified themselves as diverse indicated higher trait-BIS values than men (β = 0.18, *p* < 0.01), whereas no gender differences were observed for trait-BAS (β = 0.01, *p* = 0.76). Finally, for the CMQ-44, women and participants who classified themselves as diverse reported higher Cognitive demand values than men (β = 0.14, *p* < 0.01). Men showed higher CMQ-44 anticipation of negative consequences values than women and individuals who classified themselves as diverse (β = –0.13, *p* < 0.05). Response adaptation was higher in female and diverse participants compared to male participants (β = 0.08, *p* < 0.05). Moreover, Performance monitoring (G) was higher in male individuals compared to female and diverse participants (β = –0.24, *p* < 0.01). No significant gender differences were observed for CMQ-44 uncertainty of reinforcement (β = 0.04, *p* = 0.27). For the CMQ-28, gender differences were not robust compared with CMQ-44 (Cognitive demand: β = –0.02, *p* = 0.78; Anticipation of negative consequences: β = 0.07, *p* = 0.13; Response adaptation: β = 0.04, *p* = 0.37). In contrast to the CMQ-44, CMQ-28 uncertainty of reinforcement indicated higher factor scores for female and diverse participants compared to male participants (β = 0.12, *p* < 0.05). Comparable to the CMQ-44, CMQ-28 performance monitoring revealed higher path coefficient for men compared to women and diverse individuals (β = –0.23, *p* < 0.01).

### Convergent and discriminant validity along with evidence of robustness (research question 4)

We investigated evidence of convergent and discriminant validity of the bifactor CMQ-44 model based on factor scores for those CFA models of the (r)RST questionnaires that indicated the best model fit in terms of two or even three model fit indices (see Table 4). We indicate correlational results that correspond with our hypotheses (Tables 6, 7) in bold.

**TABLE 6:** Spearman rank correlations of the factor score-based trait-BIS-related scales (*N* = 1,076).

	CMQ-44: Cognitive demand	CMQ-44: Anticipation of negative consequences	CMQ-44: Response adaptation	CMQ-44: Uncertainty of reinforcement	CMQ-44: Performance monitoring
CW-4-factor model: BIS	0.15** (0.06*)	–0.19** (–0.13**)	–0.13** (–0.14**)	–0.08* (–0.11**)	–0.66** (–0.66**)
RST-PQ-4-factor model: BIS-MPI	–0.03 (–0.06(*))	–0.26** (–0.24**)	–0.13** (–0.14**)	–0.35** (–0.37**)	–0.58** (–0.59**)
RST-PQ-4-factor model: BIS-CRA	0.06 (*) (0.02)	–0.27** (–0.23**)	–0.16** (–0.17**)	–0.25** (–0.27**)	–0.58** (–0.57**)
RST-PQ-4-factor model: BIS-OT	0.02 (–0.01)	–0.24** (–0.21**)	–0.15** (–0.16**)	–0.27** (–0.28**)	–0.56** (–0.57**)
RST-PQ-4-factor model: BIS-BD	–0.05 (–0.08*)	–0.22** (–0.18**)	–0.14** (–0.14**)	–0.29** (–0.30**)	–0.56** (–0.58**)
RST-Q-2-factor model: BIS	–0.05 (–0.09*)	–0.26** (–0.21**)	–0.12** (–0.12**)	–0.42** (–0.44**)	–0.42** (–0.64**)

(*)*p* ≤ 0.10, *p < 0.05, **p < 0.01, ***p < 0.01, two-tailed. All *p*-values are given two-tailed. Partial correlations controlled for gender (male, female, diverse) are reported in purpose of robustness in parentheses with a sample size of *N* = 1,073. Correlations in bold are in accordance with predictions (Table 2). Correlations among the Carver-White BIS/BAS scales, the RST-PQ and the rRST-Q can be found in the Supplementary Tables 5–7.

**TABLE 7:** Spearman rank correlations of the factor score-based trait-BAS-related scales (*N* = 1,076).

	CMQ-44: Cognitive demand	CMQ-44: Anticipation of negative consequences	CMQ-44: Response adaptation	CMQ-44: Uncertainty of reinforcement	CMQ-44: Performance monitoring
CW-4-factor model: BAS-RR	0.28** (0.23**)	–0.14** (–0.15**)	–0.06(*) (–0.06(*))	0.12** (0.07*)	0.12** (0.17**)
CW-4-factor model: BAS-FS	0.20** (0.16**)	–0.07* (–0.10**)	**0.04** **(0.04)**	0.10* (0.03)	0.35** (0.40**)
CW-4-factor model: BAS-D	0.25** (0.21**)	–0.11** (–0.12**)	–0.06* (–0.06*)	0.15** (0.10**)	0.25** (0.30**)
RST-PQ-4-factor model: BAS-RI	0.20** (0.18**)	**–0.05** (–0.08**)	**0.05** **(0.05)**	0.09** (0.05(*))	0.43** (0.46**)
RST-PQ-4-factor model: BAS-GDP	0.31** (0.28**)	–0.11** (–0.12**)	–0.09** (–0.09**)	0.22** (0.18**)	0.21** (0.26**)
RST-PQ-4-factor model: BAS-RR	0.16** (0.13**)	–0.16** (–0.18**)	**–0.02** **(–0.03)**	0.02 (**–**0.02)	0.24** (0.30**)
RST-PQ-4-factor model: BAS-Imp	0.02 (0.00)	–0.12** (–0.17**)	**0.03** **(0.02)**	**–**0.16** (**–**0.22**)	0.23** (0.23**)
rRST-Q-2-factor model: BAS	0.16** (0.13**)	–0.07* (–0.10**)	**0.02** **(0.02)**	0.09* (0.05(*))	0.51** (0.54**)

(*)*p* ≤ 0.10, *p < 0.05, **p < 0.01, ***p < 0.01, two-tailed. All *p*-values are given two-tailed. Partial correlations controlled for gender (male, female, diverse) are reported in purpose of robustness in parentheses with a sample size of *N* = 1,073. Correlations in bold are in accordance with *a priori* predictions (Table 2). Correlations among the Carver-White BIS/BAS scales, the RST-PQ and the rRST-Q can be found in the Supplementary Tables 5–7.

Cognitive demand correlated positively exclusively with the Carver-White BIS factor score (Table 6). Contrary to prediction (Table 2), CMQ-44 anticipation of negative consequences, CMQ-44 response adaptation, CMQ-44 uncertainty of reinforcement, and CMQ-44 performance monitoring correlate negatively with the factor scores of the other (r)RST questionnaires indicating that these CMQ-44 factors measure different contents of trait-BIS that are not represented in the other (r)RST trait-BIS factors (Table 6). The negative inter-correlations of CMQ-44-anticipation of negative consequences, CMQ-44 response adaptation, CMQ-44 uncertainty of reinforcement, CMQ-44 performance monitoring indicate that higher CMQ-44 factor scores go along with lower (r)RST factor scores of the Carver-White BIS, the RST-PQ BIS and the rRST-Q BIS factor scores. That is, the CMQ-44 factors (except cognitive demand) do not just measure preparations of behavioral inhibition. CMQ-44 anticipation of negative consequences (ANC), response adaptation (RA), uncertainty of reinforcement (UR), and performance monitoring (G) rather measure cognitive-motivational weigh-offs prior to behavioral withdrawal. Thus, the three primary-order factors (ANC, RA, UR) and G of the CMQ-44 provide psychometric measures that are promising for investigating information processing steps before checking mode switches to control mode of the BIS and behavioral withdrawal related to Flight or Freezing (Corr, 2008). The Spearman rank correlations were performed to account for non-normality of the data. Spearman Rank correlations (Table 6) hold even when we performed partial correlations controlling for gender.

We observed positive and mainly significant inter-correlations of CMQ-44 cognitive demand with the factor scores of the other trait-BAS factors revealing that CMQ-44 cognitive demand facilitates reward-related behavior (Kool et al., 2011). Negative and mainly significant Spearman rank correlations occurred for CMQ-44 anticipation of negative consequences and CMQ-44 response adaptation indicating evidence of discriminant validity. These correlations indicate that CMQ-44 ANC and RA are not identical to BAS-related behavioral approach. CMQ-44 uncertainty of reinforcement and CMQ-44 performance monitoring appeared to correlate positively and significantly with the factor scores of the other trait-BAS factors. In this respect, it is noteworthy that CMQ-44 UR is contrary to impulsive BAS-related behavior but evokes approach behavior as does CMQ-44 performance monitoring (G). It can be supposed that BAS-oriented approach tendencies of CMQ-44 UR and G might be due to preparations from checking to control model of the BIS. The Spearman Rank correlations hold when controlled for gender in partial correlations.

## Discussion

The present study investigated psychometric properties (research question 1), evidence of factorial validity of (r)RST questionnaires (research question 2), effects of measurement equivalence for the latent (r)RST factors (research question 3), evidence of convergent and discriminant validity (research question 4). Our data reveal comparable and convincing evidence for the psychometric properties of the (r)RST questionnaires. Factorial validity has been confirmed for all (r)RST questionnaires. Best model fits have been observed for the four factor models of the Carver-White BIS/BAS scales (i.e., trait-BIS and three trait-BAS subscales), the four trait-BIS and four trait-BAS factors of the RST-PQ, the two-factor model of the RST-Q and for the CMQ-44 as well as CMQ-28 bifactor models. Gender effects were found for all inserted (r)RST questionnaires limiting measurement equivalence of the latent factors. Convergent validity for CMQ-44 cognitive demand has been exclusively observed with the Carver-White trait-BIS scales. Overall, the other CMQ-44 factors (anticipation of negative consequences, response adaptation, uncertainty of negative reinforcement, performance monitoring) rather extend the previous trait-BIS and trait-BAS space.

Correlating positively with most of the previous trait-BAS factors, the CMQ-44 cognitive demand factor appeared to be a BAS-facilitating factor (Kool et al., 2011). A similar effect occurred for RST-PQ trait-BIS subscales with RST-PQ trait-BAS Impulsivity and Carver-White BAS-Reward Responsiveness (Supplementary Table 7). While anticipation of negative consequences and response adaptation revealed evidence of discriminant validity with previous trait-BAS factors, uncertainty of negative consequences and performance monitoring extend the trait-BAS space by means of mainly significant and positive inter-correlations with previous trait-BAS factors. Higher factor scores of response adaptation of the CMQ-44 can be rather interpreted as a reactive, more flexible manner to adapt behavior (Braver, 2012; Botvinick and Braver, 2015). The small and mainly negative inter-correlations of response adaptation with the previous trait-BAS factors reveal that response adaptation is not an impulsive, spontaneous behavioral tendency. It is worth noting that individuals with more intense performance monitoring show more BAS-related behavior. Moreover, participants of the present study reported higher BAS-related approach tendencies in the previous (r)RST questionnaires even when they reported about situations with more uncertainty of reinforcement. Our data suggest evidence of convergent and discriminant validity although all included questionnaires belong to the same personality theory. The present study illustrates that personality scales in the context of (r)RST establish a nomological network. Among this nomological network the (r)RST-related personality scales operationalize different more or less overlapping parts of the trait-BIS, trait-BAS and trait-FFFS continuum. These conceptual similarities and dissimilarities between (r)RST personality scales can be documented in terms of correlations (see Tables 6, 7) and might be extended by second-order factor analyses. That is, it is a strength of the present study to include those personality scales that establish the psychometric framework of more than 20 years of psychometric (r)RST research starting with the German version of the BIS/BAS scales in 2001 (Strobel et al., 2001) and continuing to the CMQ-44 published in 2021 (Leue and Beauducel, 2021). The present study illustrates for the first time a nomological network of (r)RST questionnaires which extends the quite rare examples of nomological nets given except for the Five-factor model in the field of personality research (Ziegler et al., 2013). The fact that different researchers (Carver and White, 1994; Strobel et al., 2001; Reuter et al., 2015; Corr and Cooper, 2016; Pugnaghi et al., 2018; Leue and Beauducel, 2021) could develop independently different personality questionnaires that are suitable to comprise predictions on trait-BIS, trait-BAS, and trait-FFFS indicates in an impressive manner that (r)RST has been developed to a substantiative personality theory with extensive psychometric and neuroscientific perspectives (Gray, 1970, 1987; Gray and McNaughton, 2000).

Experimental studies investigating neural activations (e.g., frontal stimulus-locked N2 component and response-locked error-related negativity component, ERN/Ne) will elucidate the contextual foundations and individual differences of the CMQ-44 factors to further our understanding on changes between checking and control mode of the BIS (Corr, 2008). For the CMQ-44, especially anticipation of negative consequences and performance monitoring were higher in men than women. As in Leue and Beauducel (2021), CMQ-44 self-reported cognitive demand was higher in female than male participants. Overall, our psychometric data suggest that gender effects at least partly modulate individual differences of BIS/BAS scores.

### Limitations and future directions

The present data motivate further research on emic and etic issues of (r)RST questionnaires in English-speaking samples. As (r)RST questionnaires have been applied in clinical samples (Farrell and Walker, 2019), forensic samples (Leue et al., 2008; Donahu and Caraballo, 2015), and in work settings (Corr et al., 2017), it would be of interest to investigate predictions of the newly validated CMQ-44 and previous (r)RST questionnaires in forensic and clinical settings. In terms of test fairness, future research might address further evidence of measurement equivalence (e.g., for age groups). To further elucidate the nomological network we should investigate the CMQ-44 factors in relation to the five-factor model, perfectionism and with regard to intelligence (Borkenau and Ostendorf, 2008; Beauducel et al., 2010; Stoeber and Corr, 2015). To elucidate the neuroscientific basis of reward-facilitating investment of cognitive demand, individual differences of CMQ-44 cognitive demand and performance monitoring should be experimentally assessed in a study measuring event-related potentials like N2, error-related negativity (ERN/Ne), and feedback negativity (FN). The items of the included questionnaires were not developed based on psychopharmacological predictors. Future research might investigate which of those items are sufficient for correlations with psychopharmacological predictors. For examples of item developments based on psychopharmacological predictions see West and Ussher (2010). Overall, based on the scale definitions presented in Table 1 and CFA evidence we argue in favor of holding all (r)RST questionnaires for future research. When researchers wish to investigate FFFS-related trait-variations RST-PQ and RST-Q (Reuter et al., 2015; Corr, 2016; Pugnaghi et al., 2018) are recommended. When determinants and behavioral consequences of conflict monitoring are the research focus CMQ-44 and CMQ-28 are promising (Leue and Beauducel, 2021). To disentangle trait-BIS or trait-BAS responses the BIS/BAS scales, the RST-PQ and the CMQ-cognitive demand scale are sufficient psychometric candidates. The Carver-White BIS/BAS scales are required to psychometrically compare results on BAS subscales (Reuter et al., 2015) and individual differences on the N2 (Leue et al., 2012, 2014, 2020). Future research on (r)RST questionnaires might also investigate other statistical models like multiple group CFAs to compare the (r)RST personality scales for configural, metric and scalar invariances and group factors like gender (Seib-Pfeifer et al., 2017; Hein et al., 2021). In the present study, we used a construct-related parceling algorithm because all included questionnaires comprise theoretically well-defined latent constructs (Sterba and Rights, 2017). “Alternative parcel allocations” might be performed such as random item-to-parcel allocations (Sterba and Rights, 2017). Those alternative allocations have not been tested in this study. A more detailed argumentation on pros and cons of item-to-parcel allocations is given in the cited studies (Matsunaga, 2008; Little et al., 2013; Marsh et al., 2013). Further research is warranted to elucidate the trait-neurotransmitter relationship especially for the newly published (r)RST questionnaires (for an example see Reuter et al., 2006). In this respect alternative item-to-parcel allocations could be tested in order to disentangle whether a theory-related item-to-parcel allocation results in more precise trait-neurotransmitter relations than more random item-to-parcel allocations which might increase parcel-allocation variability (Sterba and Rights, 2017). Moreover, studies on the trait-neurotransmitter relation would further our knowledge on the multi-trait-multi-method matrix of (r)RST-related personality questionnaires as would be other studies combining psychometric data and data from the field of personality neuroscience (DeYoung, 2010; Leue, 2015; Asendorpf et al., 2016).

## Conclusion

The present data suggest that the Carver-White BIS/BAS scales, the RST-PQ, the rRST-Q and the CMQ-44/28 are promising personality questionnaires for the (r)RST trait space with sufficient psychometric properties. Confirmatory factor models have been mainly confirmed for trait-BIS and trait-BAS. Gender effects matter for the assessment of trait-BIS and trait-BAS. We provide evidence that factor scores are a promising tool compared to unit-weighted sum scales to elucidate convergent and discriminant validity of the trait-BIS and trait-BAS factors. These data are promising to investigate changes of the BIS from checking to control (Corr, 2008), to predict individual differences of reactive and proactive cognitive control (Braver, 2012; Botvinick and Braver, 2015), and to investigate conflict monitoring and the affect signaling hypothesis (Dignath et al., 2020).

## Data availability statement

As all inventories are part of an on-going construct validation process original data, code books, or program code will be made available in PsyArxiv upon request to the first author and depending on further validation studies: https://osf.io/9vu8e/?view_only=9655b511443c4c5e95f9393fcb15622c.

## Ethics statement

The studies involving human participants were reviewed and approved by Ethics Committee of the Medical Faculty, University of Kiel, Germany. The participants provided their written informed consent to participate in this study.

## Author contributions

AL: conceptualization, data curation and collection with team support and data processing, analysis, and wrote the manuscript. UE, MR, and PC: co-authors of the previous questionnaires for measuring the personality traits of (revised) reinforcement sensitivity theory, read, and commented on the manuscript prior to submission.

## Footnotes

1
https://www.respondi.com

2
https://www.destatis.de
