# Supplementary material for: Construct validity of questionnaires for the original and revised reinforcement sensitivity theory
Source: Front Psychol. 2022 Nov 21;13:1026894. doi: 10.3389/fpsyg.2022.1026894 (PMC9720254; doi:10.3389/fpsyg.2022.1026894)
Supplement: Supplementary file 1 [file Data_Sheet_1.docx]

Supplementary Table 1. Results of the multivariate Mardia tests of normality (*N* = 1,076).

| Model | Number of parcels | Mardia |
| --- | --- | --- |
| CW-BIS/BAS:  2-factor model | 5: cwbisp1 (02r, 08, 13), cwbisp2 (16, 19, 22r, 24),  cwbasp1 (03, 04, 05, 07), cwbasp2 (09, 10, 12, 14), cwbasp3 (15, 18, 20, 21, 23) | 37.47** |
| CW-BIS/BAS:  4-factor model | 8: cwbisp1 (02r, 08, 13), cwbisp2 (16, 19, 22r, 24),  cwrrp1 (04, 07, 14), cwrrp2 (18, 23), cwfsp3 (05, 10),  cwfsp4 (15, 20), cwdrp5 (03, 09), cwdrp6 (12, 21) | 90.66** |
| RST-PQ:  2-factor model | 14: bispqp1 (01, 02, 06, 07), bispqp2 (10, 17, 18, 21), bispqp3 (29, 33, 34, 42), bispqp4 (43, 47, 49, 50), bispqp5 (55, 56, 57, 60), bispqp6 (61, 63, 64), baspqp1 (03, 04, 05, 08), baspqp2 (11, 12, 13, 14), baspqp3 (15, 16, 20, 22), baspqp4 (23, 24, 25, 26), baspqp5 (27, 28, 30, 31), baspqp6 (32, 35, 36, 37), baspqp7 (38, 40, 41, 44), baspqp8 (51, 53, 54, 65) | 238.76** |
| RST-PQ:  3-factor model | 18: bispqp1 (01, 02, 06, 07), bispqp2 (10, 17, 18, 21), bispqp3 (29, 33, 34, 42), bispqp4 (43, 47, 49, 50), bispqp5 (55, 56, 57, 60), bispqp6 (61, 63, 64), baspqp1 (03, 04, 05, 08), baspqp2 (11, 12, 13, 14), baspqp3 (15, 16, 20, 22), baspqp4 (23, 24, 25, 26), baspqp5 (27, 28, 30, 31), baspqp6 (32, 35, 36, 37), baspqp7 (38, 40, 41, 44), | 384.11** |
| Supplementary Table 1 continued. | |  |
| Model | Number of parcels | Mardia |
|  | baspqp8 (51, 53, 54, 65), ffspqp1 (19, 45, 46), ffspqp2 (52, 58), ffspqp3 (09, 39, 48), ffspqp4 (59, 62) |  |
| RST-PQ:  4-factor model for BIS and BAS | 17: bismpip1 (18, 49, 55), bismpip2 (63, 64), biscrap3 (17, 34, 42), biscrap4 (43, 47), bisbdp5 (01, 06, 07), bisbdp6 (10, 21, 60), bisotp7 (02, 29, 33, 50),  bisotp8 (56, 57, 61), basrip1 (11, 13, 14, 15), basrip2 (26, 32, 35), basgdpp3 (05, 12, 20, 31), basgdpp4 (41, 54, 65), basrrp5 (03, 04, 08), basrrp6 (16, 23, 24), basrrp7 (25, 30, 36, 37), basimp8 (22, 27, 28, 38), basimp9 (40, 44, 51, 53) | 345.58** |
| rRST-Q:  2-factor model | 5: rmbisp1 (02, 04, 07, 08), rmbisp2 (11, 21r, 23, 26), rmbisp3 (28, 29, 30),  rmbasp1 (01, 05, 12, 13)), rmbasp2 (18, 24, 25, 31) | 39.98** |
| rRST-Q:  3-factor model | 8: rmbisp1 (02, 04, 07, 08), rmbisp2 (11, 21r, 23, 26), rmbisp3 (28, 29, 30),  rmbasp1 (01, 05, 12, 13), rmbasp2 (18, 24, 25, 31), rmffsp1 (rmfb15, rmfb16, rmfb17r, rmfb22r), rmffsp2 (rmfb06, rmfb19, rmfb20), rmffsp3 (rmfb03, rmfb09r, rmfb10, rmfb14) | 92.67** |
|  |  |  |
|  |  |  |
| Supplementary Table 1 continued. | |  |
| Model | Number of parcels | Mardia |
| CMQ-44:  4-factors and bifactor 4-factors | 19: DRprime_P3 (47, 49r, 58), DUprime_P1 (14r, 19), DUprime_P2 (24, 52r), ARprime_P3 (44r, 54r), AUprime_P1 (10r, 20r, 23), | 536.49** |
|  | AUprime_P2 (36r, 59r), RAprime_P1 (02, 21r, 57r), RAprime_P2 (48, 39), RDprime_P3 (01, 53), RDprime_P4 (17r, 22), RDprime_P5 (06, 30),  RDprime_P6 (31, 35), RDprime_P7 (38, 43, 25), UAprime_P1 (04r, 07, 12r),  UAprime_P6 (40, 45, 55r), UDprime_P2 (08r, 46), UDprime_P3 (37, 16r),  UDprime_P4 (41, 27r), UDprime_P5 (50, 56) |  |
| CMQ-28:  4-factors and bifactor 4-factors | 12: DUprime28_P1 (14r, 19)  DUprime28_P2 (24, 52r), DRprime28_P3 (47, 49r, 58), ARprime28_P3 (44r, 54r), AUprime28_P1 (10r, 20r, 23),  AUprime28_P2 (36r, 59r), RDprime28_P1 (25, 30), RDprime28_P2 (35, 43), RAprime28_P3 (02, 39, 48),  UDprime28_P1 (16r, 41), UDprime28_P2 (46, 50), UAprime28_P3 (04r, 12r, 45) | 228.22** |

Supplementary Table 2. Descriptive statistics of the (r)RST subscales (*N* = 1,076).

|  | Number of items | M (SD) | Observed Range | Skewness | Kurtosis |
| --- | --- | --- | --- | --- | --- |
| CW-BIS | 7 | 2.83 (0.63) | 1.00 to 4.00 | -0.15 | -0.23 |
| CW-BAS | 13 | 3.01 (0.45) | 1.23 to 4.00 | -0.13 | 0.11 |
| CW-BAS-FS | 4 | 2.90 (0.56) | 1.00 to 4.00 | -0.28 | 0.21 |
| CW-BAS-RR | 5 | 3.14 (0.50) | 1.20 to 4.00 | -0.32 | -0.01 |
| CW-BAS-Drive | 4 | 2.96 (0.57) | 1.00 to 4.00 | -0.10 | -0.29 |
| RST-PQ-BIS | 23 | 56.01 (15.31) | 23.00 to 92.00 | 0.09 | -0.73 |
| RST-PQ-FFFS | 10 | 24.16 (6.23) | 10.00 to 40.00 | 0.09 | -0.61 |
| RST-PQ-BIS: MPI | 5 | 11.72 (3.33) | 5.00 to 20.00 | 0.10 | -0.39 |
| RST-PQ-BIS: CRA | 5 | 11.58 (3.79) | 5.00 to 20.00 | 0.11 | -0.76 |
| RST-PQ-BIS: OT | 7 | 17.44 (5.44) | 7.00 to 28.00 | 0.11 | -0.81 |
| RST-PQ-BIS: BD | 6 | 14.28 (4.54) | 6.00 to 24.00 | 0.16 | -0.77 |
| RST-PQ-BAS | 32 | 84.99 (14.76) | 40.00 to 128.00 | -0.03 | -0.08 |
| RST-PQ-BAS: RI | 7 | 18.50 (4.47) | 7.00 to 28.00 | 0.05 | -0.46 |
| RST-PQ-BAS: GDP | 7 | 20.30 (4.41) | 7.00 to 28.00 | -0.21 | -0.46 |
| RST-PQ-BAS: RR | 10 | 27.37 (5.46) | 11.00 to 40.00 | -0.11 | -0.28 |
| RST-PQ-BAS: Imp | 8 | 18.82 (4.44) | 8.00 to 32.00 | 0.04 | -0.24 |
| rRST-Q: BIS | 11 | 2.51 (0.55) | 1.00 to 3.91 | -0.10 | -0.14 |
| rRST-Q: BAS | 8 | 2.72 (0.54) | 1.00 to 4.00 | -0.03 | 0.04 |
| rRST-Q: FFFS | 11^#^ | 2.39 (0.52) | 1.00 to 4.00 | -0.10 | 0.02 |
| CMQ-44-CD | 7 | 24.32 (5.45) | 7.00 to 41.00 | -0.12 | 0.47 |
| CMQ-44-ANC | 7 | 25.59 (5.97) | 7.00 to 42.00 | -0.07 | 0.11 |
| CMQ-44-RA | 16 | 59.17 (10.93) | 22.00 to 96.00 | 0.09 | 0.55 |
| CMQ-44-UR | 14 | 52.89 (10.93) | 18.00 to 84.00 | 0.13 | 0.40 |
| CMQ-28-RA | 7 | 25.62 (6.30) | 7.00 to 42.00 | -0.04 | 0.25 |
| CMQ-28-UR | 7 | 26.30 (6.26) | 7.00 to 42.00 | 0.07 | 0.22 |

*Note.* Sum scales (RST-PQ subscales and CMQ-44) and mean scales (CW-BIS/BAS and rRST-Q subscales). CMQ-44 descriptive statistics are based on factor scores. CD = Cognitive demand. ANC = Anticipation of negative consequences. RA = response adaptation. UR = uncertainty of reinforcement. # item 27 has been excluded from further statistical analysis because of a negative part-whole corrected item total correlation. CW-BAS-FS = Carver-White BAS subscale Fun Seeking. CW-BAS-RR = Carver-White BAS subscale Reward Responsiveness. RST-PQ-BIS: MPI = RST-PQ BIS subscale Motor Planning Interruption. RST-PQ-BIS: CRA = RST-PQ BIS subscale Cautious Risk Assessment. RST-PQ-BIS: OT = RST-PQ BIS subscale Obsessive Thoughts. RST-PQ-BIS: BD = RST-PQ BIS subscale Behavioral Disengagement. RST-PQ-BAS: RI = RST-PQ BAS subscale Reward Interest. RST-PQ-BAS: GDP = RST-PQ BAS subscale Goal-Drive Persistence. RST-PQ-BAS: RR = RST-PQ BAS subscale Reward Reactivity. RST-PQ-BAS: Imp = RST-PQ BAS subscale Impulsivity.

Supplementary Table 3. Internal consistency coefficients of the (r)RST subscales (*N* = 1,076).

|  | Number of items | Cronbach’s Alpha | Split-half Reliability:  1^st^ vs. 2^nd^ half | Split-half Reliability: Odd vs. even |  |
| --- | --- | --- | --- | --- | --- |
| CW-BIS | 7 | *.85* | *.86* | **.92** |  |
| CW-BAS | 13 | *.85* | *.85* | *.83* |  |
| CW-BAS-FS | 4 | .70 | .66 | .76 |  |
| CW-BAS-RR | 5 | .70 | .71 | .73 |  |
| CW-BAS-Drive | 4 | .76 | *.82* | .79 |  |
| RST-PQ-BIS: MPI | 5 | .77 | .76 | .76 |  |
| RST-PQ-BIS: CRA | 5 | *.82* | *.84* | *.81* |  |
| RST-PQ-BIS: OT | 7 | **.90** | *.88* | **.91** |  |
| RST-PQ-BIS: BD | 6 | *.86* | *.87* | *.89* |  |
| RST-PQ-BAS: RI | 7 | *.84* | *.80* | *.87* |  |
| RST-PQ-BAS: GDP | 7 | *.85* | *.84* | *.83* |  |
| RST-PQ-BAS: RR | 10 | *.81* | .74 | *.82* |  |
| RST-PQ-BAS: Imp | 8 | *.71* | .68 | *.75* |  |
| RST-PQ-BIS | 23 | **.95** | **.94** | **.95** |  |
| RST-PQ-BAS | 32 | **.91** | *.88* | **.93** |  |
| RST-PQ-FFFS | 10 | *.79* | .79 | *.81* |  |

Supplementary Table 3. continued.

|  | Number of items | | | Cronbach’s Alpha | Split-half Reliability:  1^st^ vs. 2^nd^ half | | Split-half Reliability: Odd vs. even |  |
| --- | --- | --- | --- | --- | --- | --- | --- | --- |
| Reuter-Montag: BIS | | 11 | *.87* | | | *.88* | *.87* |  |
| Reuter-Montag: BAS | 8 | | | *.83* | *.82* | | *.80* |  |
| Reuter-Montag: FFFS | 11^#^ | | | *.82* | *.77* | | *.87* |  |
| CMQ-44-CD | 7 | | | .77 | .77 | | *.84* |  |
| CMQ-44-ANC | 7 | | | *.82* | *.82* | | *.82* |  |
| CMQ-44-RA | 16 | | | *.88* | *.88* | | **.91** |  |
| CMQ-44-UR | 14 | | | **.90** | *.84* | | *.88* |  |
| CMQ-28-CD | 7 | | | .77 | .77 | | *.84* |  |
| CMQ-28-ANC | 7 | | | *.82* | *.82* | | *.82* |  |
| CMQ-28-RA | 7 | | | *.89* | *.89* | | *.86* |  |
| CMQ-28-UR | 7 | | | *.86* | *.88* | | *.89* |  |

*Note.* Reliabilities were computed based on item parcels. # item 27 of the rRST-Q has been excluded from further statistical analysis because of a negative part-whole item total correlation.

Supplementary Table 4. Standardized factor loadings (STDYX) of the Bifactor MIMIC model of the CMQ-28 (*N* = 1,076).

| Parcel | Factor loadings |
| --- | --- |
|  | First-order trait factors |
| *Cognitive demand* |  |
| DU_P1 (14r, 19) | .12 |
| DU_P2 (24, 52r) | .19* |
| DR_P3 (47, 49r, 58) | .24* |
| UD_P1 (16r, 41) | -.06 |
| UD_P2 (46, 50) | .32(*) |
| RD_P1 (25, 30) | .07(*) |
| RD_P2 (35, 43) | -.01 |
| *Anticipation of negative consequences* |  |
| AU_P1 (10r, 20r, 23) | .03 |
| AU_P2 (36r, 59r) | .30** |
| AR_P3 (44r, 54r) | .46** |
| RA_P3 (02, 39, 48) | -.23** |
| UA_P3 (04r, 12r, 45) | .14** |
| *Response adaptation* |  |
| DR_P3 (47, 49r, 58) | -.18** |
| AR_P3 (44r, 54r) | -.24** |
| RD_P1 (25, 30) | .43** |
| RD_P2 (35, 43) | .53** |
| RA_P3 (02, 39, 48) | .22** |
|  |  |

Supplementary Table 4 continued.

| Parcel | Factor loadings |
| --- | --- |
|  | First-order trait factors |
|  |  |
| *Uncertainty of reinforcement* |  |
| DU_P1 (14r, 19) | .26** |
| DU_P2 (24, 52r) | .24** |
| AU_P1 (10r, 20r, 23) | .10(*) |
| UD_P1 (16r, 41) | .28** |
| UD_P2 (46, 50) | -.09 |
| *G: Performance monitoring* | Second-order trait factors |
| DU_P1 (14r, 19) | .09* |
| DU_P2 (24, 52r) | .80** |
| DR_P3 (47, 49r, 58) | -.69** |
| AR_P3 (44r, 54r) | -.72** |
| AU_P1 (10r, 20r, 23) | -.80** |
| AU_P2 (36r, 59r) | -.58** |
| RD_P1 (25, 30) | .72** |
| RD_P2 (35, 43) | .73** |
| RA_P3 (02, 39, 48) | .80** |
| UD_P1 (16r, 41) | .83** |
| UD_P2 (46, 50) | .81** |
| UA_P3 (04r, 12r, 45) | .84** |

Note. ** *p* < .01, * *p* < .05, (*) *p* < .10 (all *p*-values are reported two-tailed).

Supplementary Table 5. Spearman-Rank correlations among the factor score-based Carver-White BIS scale, RST-PQ BIS scales,

and rRST-Q BIS scales (*N* = 1,076).

|  | CW-4-factor model: BIS | RST-PQ-4-factor model: BIS-MPI | RST-PQ-4-factor model: BIS-CRA | RST-PQ-4-factor model: BIS-OT | RST-PQ-4-factor model: BIS-BD | rRST-Q-2-factor model: BIS |
| --- | --- | --- | --- | --- | --- | --- |
| CW-4-factor model: BIS | -- |  |  |  |  |  |
| RST-PQ-4-factor model: BIS-MPI | .67**  (.68**) | -- |  |  |  |  |
| RST-PQ-4-factor model: BIS-CRA | .72**  (.71**) | .93**  (.93**) | -- |  |  |  |
| RST-PQ-4-factor model: BIS-OT | .68**  (.69**) | .92**  (.91**) | .99**  (.99**) | -- |  |  |
| RST-PQ-4-factor model: BIS-BD | .63**  (.64**) | .90**  (.89**) | .90**  (.90**) | .94**  (.94**) | -- |  |
| rRST-Q-2-factor model: BIS | .62**  (.65**) | .78**  (.80**) | .72**  (.73**) | .72**  (.73**) | .72**  (.73**) | -- |

Note. ** *p* < .01, * *p* < .05, (*) *p* < .10 (all *p*-values are reported two-tailed). Partial correlations controlled for gender

(male, female, diverse) are reported in purpose of robustness in parentheses with a sample size of *N* = 1,073.

Explanations of the abbreviations are related to Table 4 presented in the manuscript: CW-4-factor model: BIS =

Carver-White 4-factor model trait-BIS subscale. RST-PQ-4-factor model: BIS-MPI = Reinforcement Sensitivity Theory –

Personality Questionnaire 4-factor model including 4 trait-BIS subscales; BIS-MPI = Trait-BIS – Motor Planning

Interruption. BIS-CRA = trait-BIS – Cautious Risk Assessment. BIS-OT = trait-BIS – Obsessive Thoughts. BIS-BD =

trait-BIS – Behavioral Disengagement. rRST-Q-2-factor model: BIS = revised Reinforcement Sensitivity Theory Questionnaire,

2-factor model, trait-BIS subscale.

Supplementary Table 6. Spearman-Rank correlations among the factor score-based Carver-White BAS subscales, RST-PQ BAS subscales, and the rRST-Q BAS subscale (*N* = 1,076).

|  | CW-4-factor model: BAS-RR | CW-4-factor model: BAS-FS | CW-4-factor model: BAS-D | RST-PQ-4-factor model: BAS-RI | RST-PQ-4-factor model: BAS-GDP | RST-PQ-4-factor model: BAS-RR | RST-PQ-4-factor model: BAS-Imp | rRST-Q-2-factor model:  BAS |
| --- | --- | --- | --- | --- | --- | --- | --- | --- |
| CW-4-factor model: BAS-RR | -- |  |  |  |  |  |  |  |
| CW-4-factor model: BAS-FS | .81**  (.83**) | -- |  |  |  |  |  |  |
| CW-4-factor model: BAS-D | .87**  (.88**) | .73**  (.74**) | -- |  |  |  |  |  |
| RST-PQ-4-factor model: BAS-RI | .59**  (.62**) | .70**  (.72**) | .61**  (.63**) | -- |  |  |  |  |
| RST-PQ-4-factor model: BAS-GDP | .65**  (.65**) | .53**  (.53**) | .74**  (.74**) | .69**  (.71**) | -- |  |  |  |
| RST-PQ-4-factor model: BAS-RR | .66**  (.67**) | .60**  (.63**) | .59**  (.61**) | .73**  (.76**) | .59**  (.61**) | -- |  |  |
| RST-PQ-4-factor model: BAS-Imp | .47**  (.51**) | .55**  (.58**) | .41**  (.43**) | .71**  (.72**) | .32**  (.34**) | .72**  (.75**) | -- |  |
| rRST-Q-2-factor model: BAS | .60**  (.64**) | .70**  (.73**) | .63**  (.66**) | .73**  (.76**) | .54**  (.56**) | .62**  (.66**) | .56**  (.59**) | -- |

Note. ** *p* < .01, * *p* < .05, (*) *p* < .10 (all *p*-values are reported two-tailed). Partial correlations controlled for gender (male, female, diverse) are reported in purpose of robustness in parentheses with a sample size of *N* = 1,073. Explanations of the abbreviations are related to Table 4 presented in the manuscript: CW-4-factor model: BAS-RR = Carver-White 4-factor model trait-BAS-Reward Responsiveness subscale. BAS-FS = trait-BAS-Fun Seeking. BAS-D = trait-BAS-Drive. RST-PQ-4-factor model: BAS-RI = Reinforcement Sensitivity Theory - Personality Questionnaire, 4-factor model, trait-BAS-Reward Interest. BAS-GDP = trait-BAS-Goal Drive Persistence. BAS-RR = trait-BAS-Reward Reactivity. BAS-Imp = trait-BAS-Impulsivity. rRST-Q-2-factor model: BAS = revised Reinforcement Sensitivity Theory – Questionnaire, 2 factor model, trait-BAS subscale.

Supplementary Table 7. Spearman-Rank correlations among the factor score-based Carver-White BIS/BAS scales, RST-PQ scales, and rRST-Q scales (*N* = 1,076).

|  | CW-4-factor model: BAS-RR | CW-4-factor model: BAS-  FS | CW-4-factor model: BAS-D | RST-PQ-4-factor model:  BAS-RI | RST-PQ-4-factor model: BAS-GDP | RST-PQ-4-factor model: BAS-RR | RST-PQ-4-factor model: BAS-Imp | rRST-Q-2-factor model: BAS |
| --- | --- | --- | --- | --- | --- | --- | --- | --- |
| CW-4-factor model: BIS | .21**  (.15**) | -.18**  (-.25**) | .01  (-.06*) | -.25**  (-.29**) | .01  (-.06) | .00  (-.06(*)) | -.04  (-.03) | -.27**  (-.33**) |
| RST-PQ-4-factor model: BIS-MPI | .06  (.05) | -.16**  (-.17**) | -.09**  (-.11**) | -.20**  (-.21**) | -.08**  (-.10**) | .03  (.02) | .19**  (.23**) | -.29**  (-.31**) |
| RST-PQ-4-factor model: BIS-CRA | .14**  (.13**) | -.13**  (-.15**) | .02  (.00) | -.16**  (-.17**) | .08**  (.07*) | .06**  (.04) | .16**  (.20**) | -.23**  (-.25**) |
| RST-PQ-4-factor model: BIS-OT | .08**  (.07) | -.17**  (-.18**) | -.03  (-.05(*)) | -.20**  (-.22**) | .01  (-.01) | -.02  (-.04) | .17**  (.19**) | -.26**  (-.28**) |
| RST-PQ-4-factor model: BIS-BD | -.03  (-.05(*)) | -.23**  (-.26**) | -.14**  (-.17**) | -.31**  (-.35**) | -.12**  (-.15**) | -.15**  (-.18**) | .11**  (.13**) | -.36**  (-.39**) |
| rRST-Q-2-factor model: BIS | -.02  (-.05) | -.21**  (-.24**) | -.17**  (-.20**) | -.29**  (-.32**) | -.19**  (-.23**) | -.10**  (-.12**) | .03  (.06*) | -.36**  (-.38**) |

Note. ** *p* < .01, * *p* < .05, (*) *p* < .10 (all *p*-values are reported two-tailed). Partial correlations controlled for gender (male, female, diverse) are reported in purpose of robustness in parentheses with a sample size of *N* = 1,073. Abbreviations are reported in the Notes auf Tables S5 and S6.


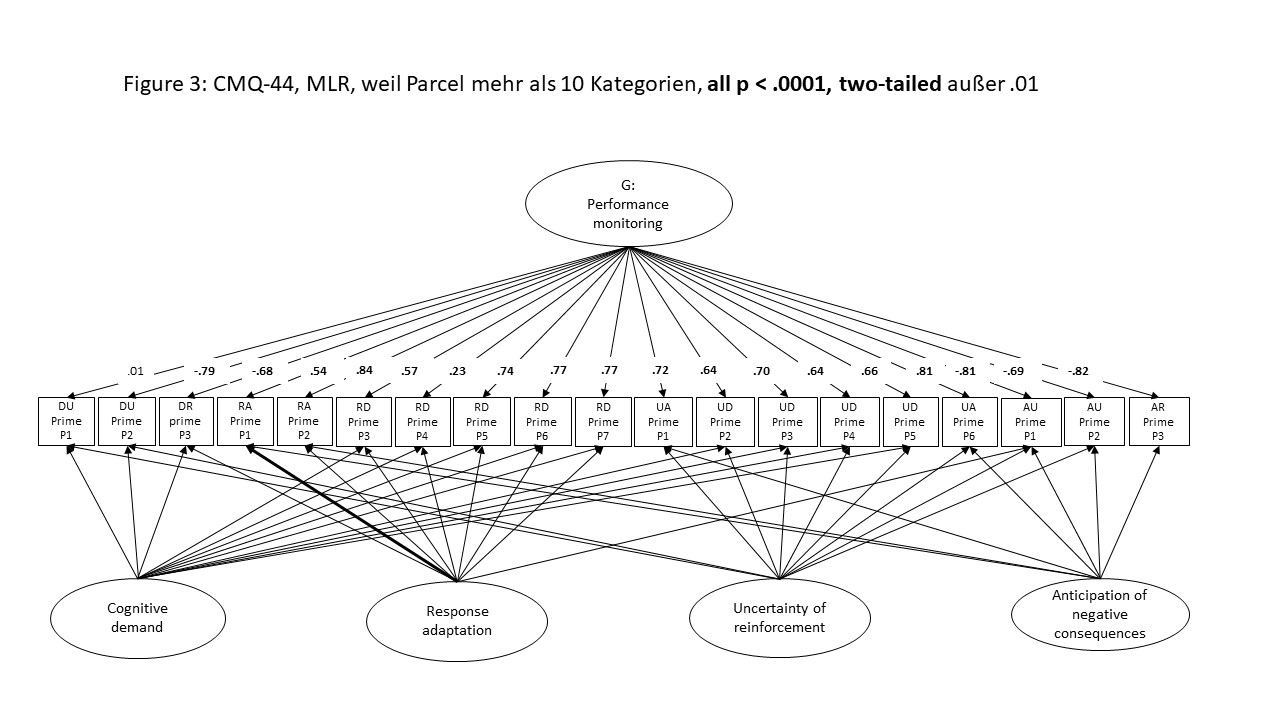


Supplementary Figure 1. Bifactor MIMIC model for the CMQ-44. Table 5 summarizes the standardized factor loadings (STDYX). For the second-order factor G factor loadings given in bold have a significance level of *p* < .0001, two-tailed.


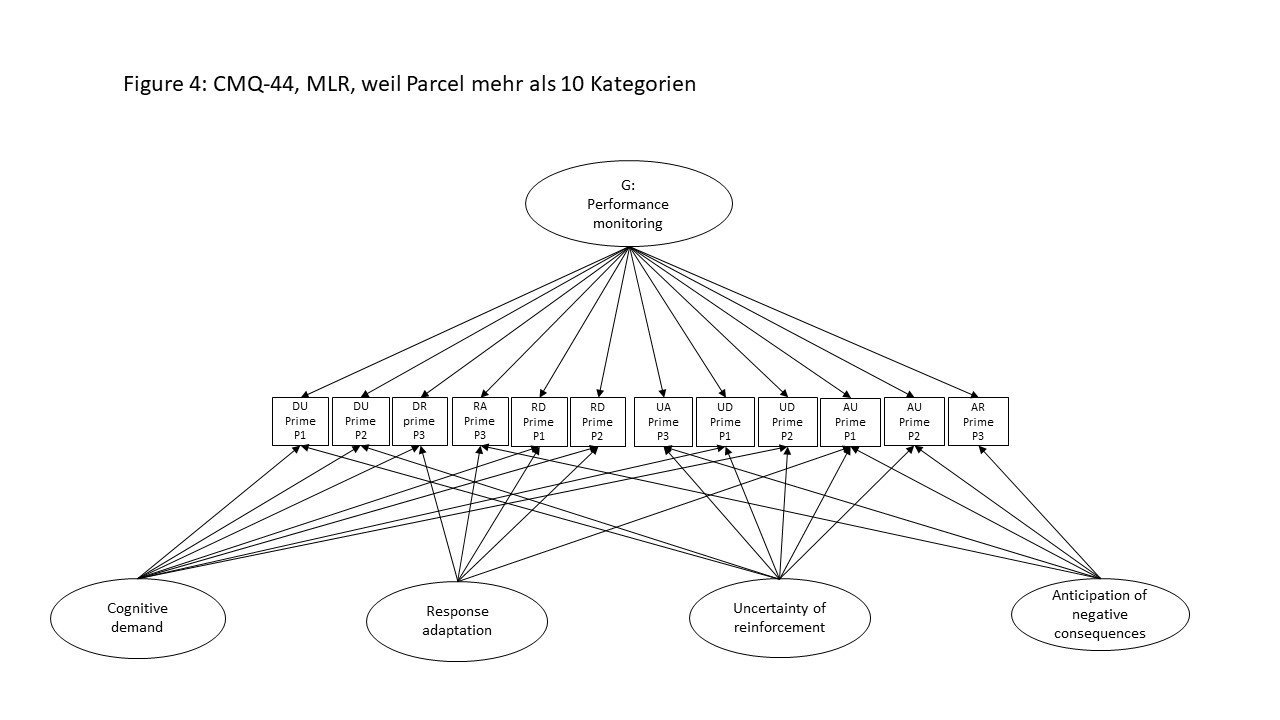


Figure S2. Bifactor MIMIC model for the CMQ-28. Please see Table S3 for standardized factor loadings (STDYX) with * *p* < .05, two-tailed. ** *p* < .01, two-tailed. (*) *p* < .10, two-tailed.
